# Inhibition of spring viraemia of carp virus replication in an *Epithelioma papulosum cyprini* cell line by RNAi

**DOI:** 10.1111/jfd.12227

**Published:** 2014-01-27

**Authors:** M Gotesman, H Soliman, R Besch, M El-Matbouli

**Affiliations:** 1 Clinical Division of Fish Medicine, University of Veterinary MedicineVienna, Austria; 2 Fish Medicine and Management, Faculty of Veterinary Medicine, University of AssiutAssiut, Egypt; 3 Clinic and Policlinic for Dermatology and Allergology, Department of Dermatology, Ludwig-Maximilian UniversityMunich, Germany

**Keywords:** nucleoprotein, phosphoprotein, real-time quantitative reverse-transcription PCR, *Rhabdoviridae*, siRNA

## Abstract

Spring viraemia of carp virus (SVCV) is an aetiological agent of a serious disease affecting carp farms in Europe and is a member of the *Rhabdoviridae* family of viruses. The genome of SVCV codes for five proteins: nucleoprotein (N), phosphoprotein (P), matrix protein (M), glycoprotein (G) and RNA-dependent RNA polymerase (L). RNA-mediated interference (RNAi) by small interfering RNAs (siRNAs) is a powerful tool to inhibit gene transcription and is used to study genes important for viral replication. In previous studies regarding another member of *Rhabdoviridae*, siRNA inhibition of the rabies virus nucleoprotein gene provided *in vitro* and *in vivo* protection against rabies. In this study, synthetic siRNA molecules were designed to target SVCV-N and SVCV-P transcripts to inhibit SVCV replication and were tested in an *epithelioma papulosum cyprini* (EPC) cell line. Inhibition of gene transcription was measured by real-time quantitative reverse-transcription PCR (RT-qPCR). The efficacy of using siRNA for inhibition of viral replication was analysed by RT-qPCR measurement of a reporter gene (glycoprotein) expression and by virus endpoint titration. Inhibition of nucleoprotein and phosphoprotein gene expression by siRNA reduced SVCV replication. However, use of tandem siRNAs that target phosphoprotein and nucleoprotein worked best at reducing SVCV replication.

## Introduction

Spring viraemia of carp virus (SVCV) was initially isolated and characterized as the aetiological agent of a serious disease affecting carp farms of Central and Eastern Europe (Fijan *et al*. 1971; Ahne 1978). Since then, the virus has been reported in Russia (Oreshkova *et al*. 1999) and detected in tissue samples originating from Hungary and Czech Republic (Koutná *et al*. 2003). Recently, the virus has been isolated in China (Teng *et al*. 2007), and SVCV-associated mass mortality events were reported in the United States (Dikkeboom *et al*. 2004; Phelps *et al*. 2012). SVCV is a member of the *Rhabdoviridae* family of viruses; in addition to carp, other Rhabdoviruses infect multiple forms of fresh water fish and marine life (Talbi *et al*. 2011).

The genome of SVCV consists of a single-stranded, negative-sense RNA molecule of 11019 nucleotide bases and codes for five proteins from 3′ to 5′ direction (Hoffmann, Schütze & Mettenleiter 2002): nucleoprotein (N), phosphoprotein (P), matrix protein (M), glycoprotein (G) and RNA-dependent RNA polymerase (L). All five viral proteins L, G, P, N and M with respective masses of 238, 69, 50, 47 and 25 kDa, have been visualized by Coomassie staining, and SVCV proteins G, P and N have also been recognized by different monoclonal antibodies, which are each specific for the respective protein (Chen *et al*. 2008). SVCV-G shares high protein sequence homology to other *Rhabdoviridae* glycoproteins (Johnson *et al*. 1999). The N and P proteins of SVCV are associated with the viral genome and play an important role in viral transcription and replication (Roy 1981). In the influenza virus, nucleoprotein interacts with viral polymerase during transcription and replication (Portela & Digard 2002) and binds to single-stranded RNA to stabilize and package viral template (Coloma *et al*. 2009). In vesicular stomatitis virus, phosphoprotein serves as a bridge between RNA template and the polymerase via the N-domain and also functions in regulating the processivity of the polymerase using a 26-amino acid stimulatory domain (Rahmeh *et al*. 2012).

RNA-mediated interference (RNAi) by double-stranded RNA (dsRNA) was originally demonstrated in *Caenorhabditis elegans* in studying the twitching phenotype caused by the disruption of a non-essential myofilament protein termed ‘*unc-22*’ by Fire *et al*. (1998). Since then, the mechanism and machinery for small non-coding RNA to function in RNAi has been worked out in great detail for a myriad of organisms in terms of gene silencing of either endogenous- or exogenous-derived transcripts by the respective microRNA (miRNA) pathway (Stefani & Slack 2008) and small interfering (siRNA) pathway (Sifuentes-Romero, Milton & García-Gasca 2011), and for DNA elimination by the scan RNA (scnRNA) pathway (Mochizuki 2010). During gene silencing of exogenous transcripts, the RNA-induced silencing complex (RISC) converts long dsRNA transcripts into short double-stranded RNA oligonucleotides (21–25nt), which later guide the RNAi machinery to degrade targeted genes via antisense recognition (Kim *et al*. 2006). The cleavage of long dsRNAs into short oligonucleotides is processed by an RNase III enzyme termed Dicer, and thereafter, in some organisms, the oligo is incorporated into either the miRNA or siRNA pathway, depending on which Dicer homolog processed the oligo (Lee *et al*. 2004). Argonaute, which was initially indentified in *Arabidopsis thaliana*, along with the piwi subfamily are the main protein components of RISC used in many organisms to determine the post-transcriptional gene-silencing pathway (Mallory & Vaucheret 2010). Currently, RNAi-based technology has been refined to be used to inhibit a gene of interest by synthetically generated double-stranded RNA (dsRNA) oligonucleotides that have antisense recognition to targeted genes. Transfection of these synthetic small interfering RNAs (siRNAs) into cells leads to silencing of the targeted transcript. This technique is used in a myriad of applications that include gene function analysis and therapeutics (Gavrilov & Saltzman 2012) and has been suggested to be useful in fish medicine to combat various types of diseases that are caused by viral and parasitic agents (Lima, Harris & Cook 2013).

Previous attempts at protecting carp from SVCV using a DNA vaccine that drives the expression of the SVCV-G protein provided varying degree of protection from SVCV-induced mortality (Kanellos *et al*. 2006; Emmenegger & Kurath 2008). However, DNA vaccines must be administered prior to infection to allow for a robust immune response to develop and cannot be used to treat infected fish. Conversely, RNAi-based strategy may yield higher *in vivo* protection to SVCV than DNA vaccines and may lead to a therapy strategy to save SVCV-infected fish. In studies that use siRNA to target the mammalian-specific viruses of the *Rhabdoviridae* family (RABV), silencing the nucleoprotein resulted *in vitro* and *in vivo* protection from infection with the rabies virus (Gupta *et al*. 2012; Yang *et al*. 2012). This current study tests the feasibility of using synthetic dsRNA oligonucleotides (21 nt) that target SVCV-N and SVCV-P genes to inhibit *in vitro* SVCV replication utilizing in an *epithelioma papulosum cyprini* (EPC) cell line. The findings from this study may ultimately lead to a novel method to protect fish from SVCV and a strategy to treat SVCV-infected fish. The siRNA molecules were designed to target SVCV-N and SVCV-P transcripts. The knock-down (KD) efficiency of the siRNA molecules was measured by real-time quantitative reverse-transcription PCR (RT-qPCR). The inhibition of viral replication was also measured by quantifying the expression of a reporter gene (glycoprotein) by RT-qPCR as performed in similar studies (Ruiz *et al*. 2009) and by virus endpoint titration in cell culture (Reed & Muench 1938).

## Materials and methods

### Cell culture

The *epithelioma papulosum cyprini* (EPC-173) was propagated in ZB5 medium: MEM Earle's salts 480 mL L^−1^ (Invitrogen), MEM, Hank's salt 480 mL L^−1^ (Invitrogen), Gibco non-essential amino acids 10 mL L^−1^ (Invitrogen), Na-pyruvate 120 mg L^−1^ with 10% per volume foetal bovine serum (FBS) and antibiotic/antimycotic mix (Sigma-Aldrich). For cell culture passaging, EPC cells (after reaching a minimum of 70% confluence) were split in an 1:3 ratio by trypsinization and seeded in fresh ∼5 mL medium in 25-cm^2^ cell culture flasks (Sigma-Aldrich), at initial concentrations of approximately 2.4 × 10^5^ cells mL^−1^ at 20 °C. For experimental conditions with siRNA, EPC cells (after reaching a minimum of 70% confluence) were split in a 1:2 ratio and seeded on 24-well cell culture plates (Sigma-Aldrich), at initial concentrations of approximately 3.6 × 10^5^ mL^−1^ cells in 1 mL of media per well, at 20 °C.

### Tissue extraction

Tissues (kidney and spleen) from a carp confirmed by PCR and sequencing to be infected with SVCV were homogenized in minimum essential medium. An aliquot of the homogenate was used for propagation of SVCV in the aforementioned EPC cell line. After propagation, total RNA extracted from the supernatant was later used as a positive (POS) control for SVCV replication.

### Viral propagation

Two days post-seeding, 25-cm^2^ cell culture flasks were emptied of media and covered with 0.5 mL of previously propagated SVCV aliquots. After 1 h of incubation at 20 °C, the flask culture was replenished with an additional 5 mL of ZB5 medium and further incubated at 20 °C for an additional 2 days. The resulting supernatant was collected into aliquots and stored at −80 °C.

### Viral titration

Fresh EPC cells were seeded on Cellstar 96-well plate (Greiner Bio-One), at initial concentrations of approximately 2.4 × 10^5^ mL^−1^ cells in 225 μL of medium, per well at 20 °C. Two days post-seeding, 25-μL aliquots of 10× serial dilutions of SVCV supernatant (as previously described) were added to each well. Cytopathic effect (CPE) was observed 2 days after seeding, but wells were scored 4 days after seeding. Reed & Muench's (1938) method was used to calculate Tissue Culture Infective Dose 50% (TCID_50_) mL^−1^.

### siRNA design

Small interfering double-stranded RNAs to target SVCV nucleoprotein and phosphoprotein genes, GenBank access number NC_002803, respective Gene ID's: 921324 and 921323, were designed using Block-iT RNAi Designer (Invitrogen) and were synthesized by Ambion (Invitrogen) to carry dTdT 3′ overhangs. Two different siRNAs were developed to target regions in the nucleoprotein gene: the first one termed ‘N’ (GGGAUAGCUUCGGACACAATT, antisense strand 5′-UUGUGUCCGAAGCUAUCCCTT) targets (GGGATAGCTTCGGACACAA), and another one termed ‘N1’ (sense strand: GCUGAUGGAAUCCCUGAUATT, antisense strand 5′-UAUCAGGGAUUCCAUCAGCTT) targets (GCTGATGGAATCCCTGATA). Similarly, two different siRNAs were developed to target regions in the phosphoprotein gene: the first one termed ‘P’ (sense strand: GGAAUCAGAUUCGGGAGAUTT, antisense strand 5′-AUCUCCCGAAUCUGAUUCCTT) targets (GGAATCAGATTCGGGAGAT), and another one termed ‘P1’ (sense strand: CCUGAUUACCUCAGAGAAATT, antisense strand 5′-UUUCUCUGAGGUAAUCAGGTT) targets (CCTGATTACCTCAGAGAAA). Two siRNAs targeting non-SVCV regions, but regions of a different fish virus (Cyprinid herpesvirus-3), were used as controls. One siRNA is termed ‘DP’ (CCUCUACAACGUGCACUUUTT, AAAGUGCACGUUGUAGAGGTT), and the other siRNA is termed ‘TK’ (UCGACGAGGGACAGUUCUUTT, AAGAACUGUCCCUCGUCGATT). Sense and antisense strands were resuspended in DEPC-treated water to obtain 20 μm (0.266 μg μL^−^^1^) solutions, heated at 90 °C for 1 min for denaturation and incubated at 37 °C for 60 min for annealing. They were next allowed to cool to room temperature and were aliquoted for use.

### siRNA application

One day after seeding EPC cells into fresh 24-well plates, siRNA was transfected according to the manufacturer's instructions in the 24-well plates. Briefly, siRNA was resuspended in separate 100 μL opti-mem® I reduced-serum medium aliquots (Invitrogen) and incubated with 1 μL Lipofectamine® LTX reagent (Invitrogen) at room temperature for 30 min, and the appropriate siRNA solution was applied into individual wells. The plates were returned to incubate at 20 °C for an additional 24 h.

### Virus application

In the first trial, following the siRNA application (the third day after seeding), medium from a plate was replaced with fresh medium supplemented with SVCV at 10^5.85^ TCID_50_ mL^−1^ returned to incubation at 20 °C. On the next day (the fourth day after seeding), the medium was also replaced with fresh medium and returned to incubation at 20 °C. The following day, media were collected and aliquoted for RNA extraction and viral titre analysis. The second trial was performed in duplicates; following the siRNA application (on the third day of the trial), medium was supplemented with SVCV to form a final concentration of 10^4.85^ TCID_50_ mL^−1^ and returned to incubate at 20 °C. Two days later, the fifth day after seeding (as performed in our initial trial), medium was collected and aliquoted for RNA extraction and viral titre analysis.

### RT-qPCR analysis

Total viral RNA was extracted from 200 μL medium fractions using the QIAamp viral RNA mini kit (Qiagen). RNA expression for released viral particles was measured in duplicates using the CFX real-time system attached to a C100 Touch thermal cycler (Bio-Rad). Primer sets coding for a region in SVCV-N gene, forward primer (FP)-AACAGCGCGTCTTACATGC and reverse primer (RP)- CTAAGGCGTAAGCCATCAGC, and coding for a region in SVCV-P gene, FP-TGAGGAGGAATGGGAATCAG and RP-AGCTGACTGTCGGGAGATGT, were used to measure RNA expression by RT-qPCR with SYBR Green I. The protocol for RT-qPCR was as follows: 50 °C for 30:00 min, 95 °C for 15:00 min, 40 cycles of 94 °C for 0:15 min, 60 °C for 1:00 min and plate read. The program was continued with a melting curve analysis: 94 °C for 1:00 min, 60 °C for 0:31 min, 70 cycles of 60 °C with a 0.5 °C ramp/cycle and plate read. TaqMan hydrolysis RT-qPCR was used in duplicates to quantify relative SVCV replication based on the Yue *et al*.'s (2008) protocol. Briefly, primers for SVCV-glycoprotein gene (FP- TGCTGTGTTGCTTGCACTTATYT, RP-TCAAACKAARGACCGCATTTCG and FAM-ATGAAGARGAGTAAACKGCCTGCAACAGA-BHQ1) were used for RT-qPCR with the following protocol: 50 °C for 30:00 min, 95 °C for 15:00 min, 40 cycles of 94 °C for 0:15 min, 60 °C for 1:00 min and plate read.

## Results

### Viral propagation

**Figure 1:**
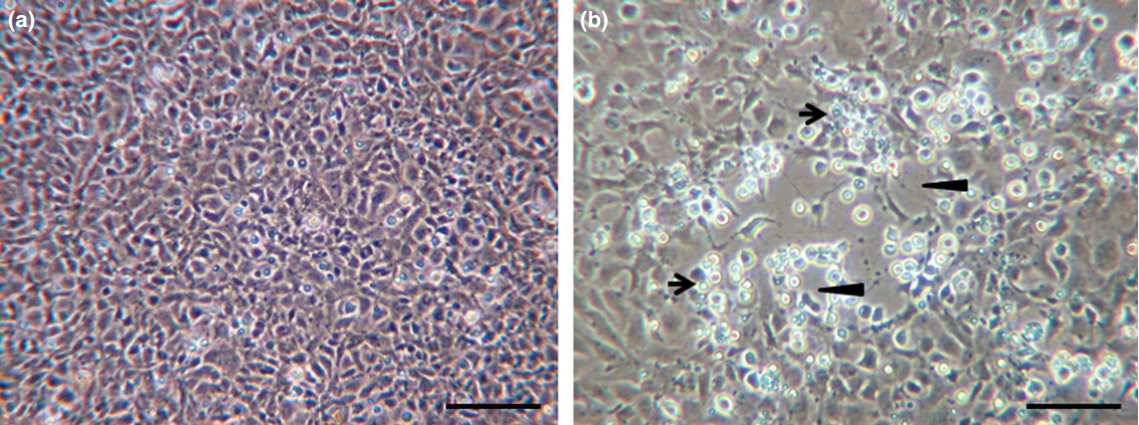
Spring viraemia of carp virus (SVCV)-induced cytopathic effect (CPE). Images of *Epithelioma papulosum cyprini (*EPC) were taken 2 days post-incubation with SVCV. (a) Untreated, control cells (b) SVCV-infected cells. Arrows point to SVCV-infected rounded-up cells and arrow heads point to SVCV-induced cell clearance. Scale bar = 50 μm.

In EPC infected with SVCV, early signs of CPE were observed 1 day post-infection (dpi) of the virus, and complete clearance of EPC cells was observed 2 dpi (Fig.1). The virus titre 2 dpi measured 10^7.85^ TCID_50_ mL^−1^. For siRNA experiments, the virus was diluted 100- or 1000-fold (v/v) to form final 10^5.85^ TCID_50_ mL^−1^ or 10^4.85^ TCID_50_ mL^−1^, respectively.

### Initial trial

**Figure 2:**
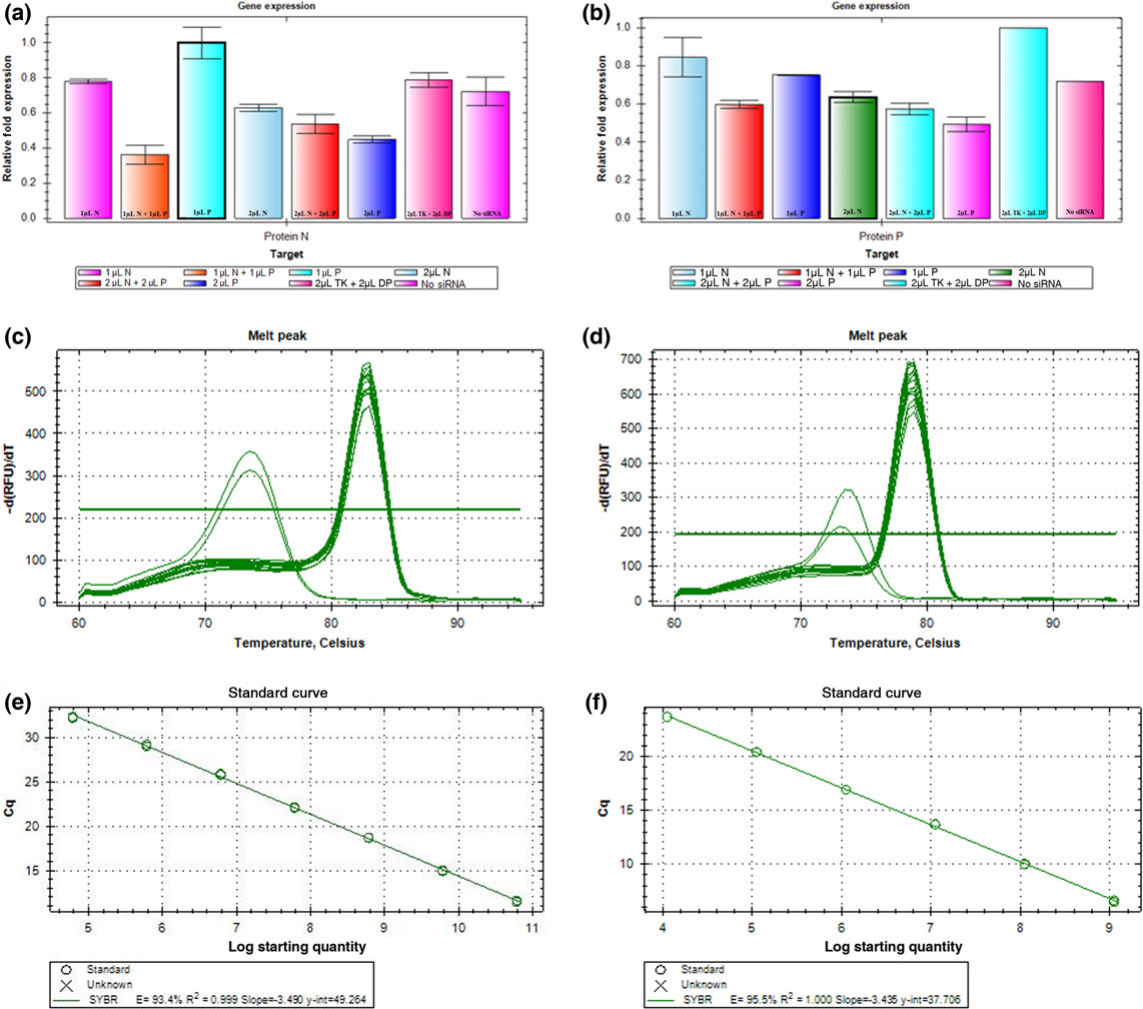
Quantification of siRNA knock-down. The efficiency of siRNA knock-down of spring viraemia of carp virus (SVCV) genes was evaluated by RT-qPCR using SYBR Green I, as described in the methods. For figures a–b, the *y*-axis refers to relative gene copy number as compared to the sample with the highest copy number, the *x*-axis refers to the volume and type of siRNA administered, N (nucleoprotein), P (phosphoprotein), DP and TK are control sequences as described in the methods. The volume 1 μL is equivalent to 20 μm (0.266 μg μL^−1^) of siRNA duplexes, and 2 μL is 40 μm (0.532 μg μL^−1^) (a) RT-qPCR measuring nucleoprotein transcripts (b) RT-qPCR measuring phosphoprotein transcripts. For figures c–d, meltcurves for RT-qPCR measuring (c) nucleoprotein transcripts, intended product at −83 °C, primer dimers for no template control (NTC) at −73 °C (d) phosphoprotein transcripts, intended product at −79 °C, primer dimers for no template control (NTC) at −74 °C. For figures e–f, standard curves for the primers were made, (e) nucleoprotein and (f) phosphoprotein.

The efficiency of gene knock-down by small interfering RNAs for released viral particles was evaluated by RT-qPCR with SYBR Green I. Analysis of both SVCV nucleoprotein and phosphoprotein genes showed that 1 μL (0.266 μg μL^−1^) treatment of siRNA per 1-mL wells for a final concentration of 20 nm of siRNA per well targeting either SVCV-N or SVCV-P transcripts inhibited expression of those respective transcripts nominally as compared to untreated or control siRNA (Fig.2a–b). Addition of 2 μL (0.532 μg μL^−1^) of either siRNAs for a 40 nm final concentration or the simultaneous addition of 1 or 2 μL of each siRNAs for respective final concentrations of 40 or 80 nm of combined siRNA per well was more effective at decreasing the expression of SVCV-N and SVCV-P transcripts. The reduction in mRNA transcript levels for the nucleoprotein gene by doubling the amount of siRNA used resulted in decreases of 25% and 55% when siRNAs against either N or P were used, respectively. And the reduction in mRNA transcript levels for the phosphoprotein gene by doubling the amount of siRNA used resulted in decreases of 24% and 38% when siRNAs against either N or P were used, respectively. However, targeting both genes by treatment with 2 μL of each siRNA produced only a nominal difference in transcription of either N or P genes, as compared to treatment with only 1 μL of each siRNA. The melt curves of these primer sets show them to be specific for their intended targets (Fig.2c–d) and showing a lower T_m_ only in the no template control (NTC) possibly indicating the formation of primer dimers in the NTC (Bustin & Nolan 2004). Primer dimers in the NTC are a common phenomenon of qPCR with SYBR Green I and are difficult to eliminate (Ponchel *et al*. 2003). A standard curve for each primer set was tested for the detection of SVC virus and showed the primers to be efficient for the detection of the virus (Fig.2e–f).

**Figure 3:**
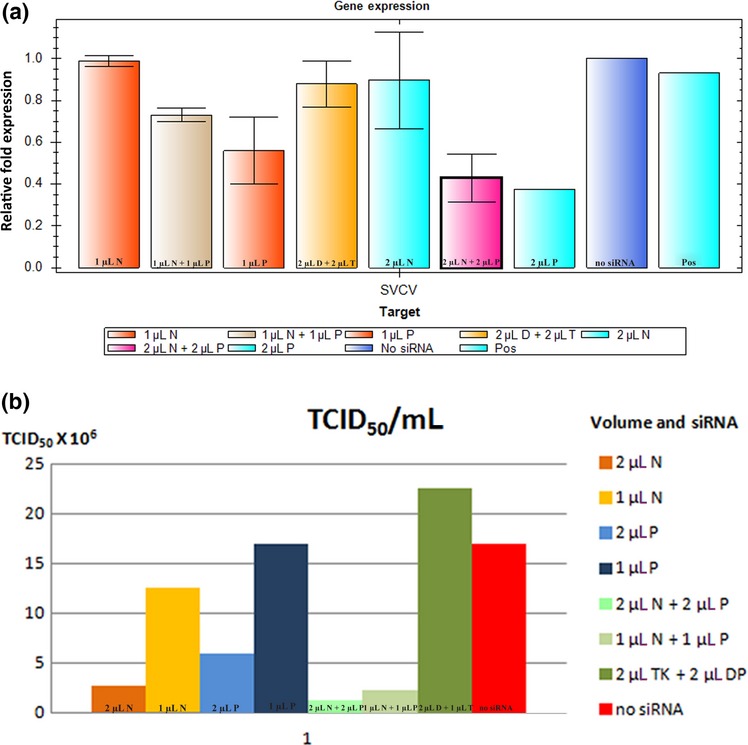
Inhibition of viral replication in the first trial. For figures a–b, TaqMan hydrolysis RT-qPCR and virus endpoint titration were used to measure the inhibition of viral replication. (a) TaqMan hydrolysis RT-qPCR, the *y*-axis refers to relative gene copy number of glycoprotein as compared to the sample with the highest copy number, the *x*-axis refers to the volume and type of siRNA administered, N (nucleoprotein), P (phosphoprotein), D and T are control sequences (DP & TK) as described in the methods, and POS (Positive Control) which was RNA extracted from the tissue of an spring viraemia of carp virus (SVCV)-infected carp. The volume 1 μL is equivalent to 20 μm (0.266 μg μL^−1^) of siRNA duplexes and 2 μL is 40 μm (0.532 μg μL^−1^). (b) Virus endpoint titration, the *y*-axis refers 10^6^ TCID_50_ mL^−1^ the coloured coated bar graphs refer to the volume and type of siRNA administered, N (nucleoprotein), P (phosphoprotein), DP and TK are control sequences as described in the methods.

According to TaqMan hydrolysis RT-qPCR, levels of SVCV-glycoprotein gene expression were similar in RNA extractions from virus propagation in either cell culture (no siRNA) or tissue extraction (POS) control, and that siRNA targeting SVCV-P was more effective than targeting SVCV-N at reducing mRNA transcripts for SVCV-G (Fig.3a). Also, the addition of 2 μL of siRNA targeting SVCV-P or the simultaneous addition of 1 or 2 μL of each siRNAs significantly decreased the expression of SVCV-G transcripts. To determine the effectiveness of siRNA knock-down to inhibit SVCV replication, viral titrations were performed from the medium collected post-siRNA treatment. In accordance with the results from RT-qPCR, treatment with 1 μL of siRNA targeting either SVCV-N or SVCV-P genes had only a minimal effect on the TICD_50_ mL^−1^ (Reed & Muench 1938) as compared to non-treatment with siRNA (Fig.3b). However, in accordance with all RT-qPCR results, treatment with 2 μL of either siRNAs or addition of 1 or 2 μL of each siRNAs significantly decreased the TICD_50_ mL^−1^, as much as 13- to 18-fold as compared to untreated sample or mistargeted siRNA, respectively.

### Second trial

**Figure 4:**
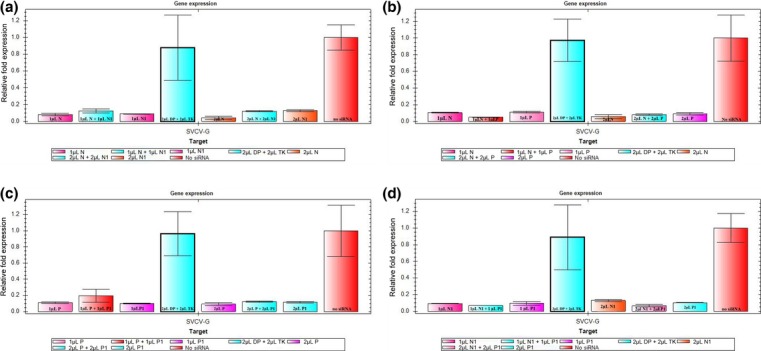
Inhibition of viral replication in the second trial. For figures a-d, TaqMan hydrolysis RT-qPCR was used to measure the inhibition of viral replication. The *y*-axis refers to relative gene copy number of glycoprotein as compared to the sample with no siRNA treatment, the *x*-axis refers to the volume and type of siRNA administered, N and N1 refer to siRNAs targeting nucleoprotein, P and P1 refer to (phosphoprotein), and DP and TK are control sequences as described in the methods. The volume 1 μL is equivalent to 20 μm (0.266 μg μL^−1^) of siRNA duplexes and 2 μL is 40 μm (0.532 μg μL^−1^).

To determine whether siRNA treatment was more effective during an infection beginning with a lower viral load, we repeated our first trial with an initial viral load concentration of 10^4.85^ TCID_50_ mL^−1^. A significant difference of reduction for glycoprotein transcription was observed in all treatments with siRNA compared with the control groups, as measured by RT-qPCR (Fig.4). As in our previous trial, the use of 2 μL of either siRNAs or addition of 1 or 2 μL of each siRNAs was most effective at reducing SVCV replication (Table1).

**Table 1:** Inhibition of viral replication by siRNA

Group	Treatment	Relative Quantity SVCV-G	10^6^ TCID_50_ mL^−1^
N	1 μL N	0.10026	5.3750
	2 μL N	0.05329	3.3155
	1 μL N1	0.10428	4.1850
	2 μL N1	0.15969	1.9800
	1 μL N + 1 μL N1	0.15658	7.1450
	2 μL N + 2 μL N1	0.11062	4.1850
P	1 μL P	0.10975	7.1450
	2 μL P	0.09270	7.7050
	1 μL P1	0.10115	5.9200
	2 μL P1	0.11570	3.8050
	1 μL P + 1 μL P1	0.19434	9.2850
	2 μL P + 2 μL P1	0.12242	6.3350
N + P	1 μL N + 1 μL P	0.04837	1.9800
	2 μL N + 2 μL P	0.07659	1.9800
	1 μL N1 + 1 μL P1	0.07530	6.9300
	2 μL N1 + 2 μL P1	0.07528	3.5900
Control	2 μL DP + 2 μL TK	0.95522	17.7050
	no siRNA	1.00000	19.7000

The inhibition of viral replication by specific siRNAs was measured by RT-qPCR of a viral reporter gene (glycoprotein) and by virus endpoint titration. The treatments were in grouped in terms of siRNA targeting nucleoprotein (N), phosphoprotein (P), a combination of the two N +P or controls. The relative quantity is given in comparison with treatment with no siRNA in the 3rd column as measured by RT-qPCR. Similarly, the 4th column shows the average of two TCID_50_ mL^−1^ trials.

## Discussion

Spring viraemia of carp virus is a major disease affecting the European carp industry (Ahne *et al*. 2002) and is most active during the spring weather, when the temperature is between 10 and 20 °C (Ahne 1986). In natural infection by SVCV, entry and early replication is via the gills, followed by dispersal via the blood stream to the kidney, liver, spleen and other major organs (Ahne 1978). Detection methods for SVCV include cell culture propagation (Ahne 1978), enzyme-linked immunosorbent assay (Rodák *et al*. 1993), competitive immunoassay (Dixon, Hattenberger-Baudouy & Way 1994) and newer methods include RT-qPCR (Yue *et al*. 2008) and visual-based methods that use gold nanoparticles (Saleh *et al*. 2012). SVCV replicates in the cytosol and both nucleoprotein and phosphoprotein are phosphorylated (Sokol & Koprowski 1975). In addition to having sites for phosphorylation, both aforementioned proteins likely interact with the RNA-dependent polymerase as described in other RNA viruses (Coloma *et al*. 2009; Rahmeh *et al*. 2012). Although several antiviral genes, as well as the interferon pathway, are upregulated during SVCV infection (Adamek *et al*. 2012), the mortality rate for young carp can reach up to 70% during spring time outbreaks (Ahne *et al*. 2002). The defensive capabilities of cells against viral infection can be enhanced using different forms of RNA that include RNAi (Porntrakulpipat *et al*. 2010; Hwang *et al*. 2012). The RNAi machinery can be induced by synthetic double-stranded RNA oligonucleotides (21–22nt), termed ‘siRNAs’ (Elbashir, Lendeckel & Tuschl 2001b). The efficiency for knock-down by siRNA duplexes ranges from 2- to 25-fold and is dependent on various factors, such as gene of target, region targeted and cell line used (Elbashir *et al*. 2001a). The siRNA knock-down (KD) of the nucleoprotein from another fish rhabdovirus, termed ‘viral hemorrhagic septicaemia virus’ (VHSV), greatly reduced clearance capacity of the virus on EPC cells (Ruiz *et al*. 2009). RNAi-mediated knock-down of other VHSV genes in EPC has also been shown to inhibit viral infection (Kim & Kim 2011; Kim *et al*. 2012). For Rabies virus, targeting nucleoprotein transcripts by siRNA showed higher inhibition than targeting the polymerase (Gupta *et al*. 2012). However, we are unaware of a study that uses RNAi to investigate the inhibition of SVCV. In this study, we designed siRNAs that target either SVCV-N or SVCV-P genes to inhibit *in vitro* SVCV replication in EPC. In addition, novel primers were developed to quantify SVCV-N or SVCV-P mRNA transcription.

The efficiency of knocking down either SVCV-N or SVCV-P transcripts in reducing viral activity was evaluated by two different methods: one that uses TaqMan hydrolysis RT-qPCR to measure the reduction of a reporter gene (glycoprotein) and another one that measures the reductivity of viral activity in cell culture (Reed & Muench 1938). Both methods showed that siRNA knock-down of either SVCV-N or SVCV-P transcripts reduced viral activity. According to the TaqMan hydrolysis RT-qPCR results, siRNA targeting of either phosphoprotein or nucleoprotein genes alone inhibited SVCV-G transcription. However, viral titration in cell culture showed a strong correlation for inhibition of virus replication in response to the amount of siRNA treatment, and the use of tandem siRNAs that target both SVCV-N and SVCV-P genes was the most effective at inhibition of SVCV replication. As opposed to dsRNA that are 30nt or longer that can activate the interferon response pathway, dsRNA that are between 21 and 25nt have been shown to target specific silencing mechanisms (Caplen *et al*. 2001). To confirm that inhibition of gene transcription and viral replication was specific to the chosen siRNAs and not a general response by the interferon or other host defence pathway, siRNAs targeting another virus were chosen as controls. The control siRNAs did not show any effects on SVCV, which suggests that the inhibition of gene transcription and viral replication was a specific response induced by the RNAi machinery.

Previously, *in vivo* application of RNAi has been shown to combat white spot syndrome virus (WSSV), an aquatic viral disease of shrimp (Sarathi *et al*. 2010). The results of this study should also be tested in an *in vivo* model to determine how well SVCV replication can be inhibited by RNA-mediated interference. Earlier studies have outlined some of the difficulties of transferring positive results for siRNA studies from *in vitro* to in *vivo* trials (Schyth 2008), such as the temporary effect of siRNAs and the activation of the interferon response pathway (Schyth, Lorenzen & Pedersen 2007). The chemical modifications of siRNAs (Schyth *et al*. 2012) or the use of longer dsRNA (27/25 mer) that is a cellular substrate target of dicer (Bohle, Lorenzen & Schyth 2011) provide for alternative methods to study the *in vivo* effects of siRNA knock-down. Although current EPC lineages are contaminated with cells from another cyprinidae, fathead minnow *Pimephales promelas* (Winton *et al*. 2010), EPC remains a current subject for the study of RNAi in fish cells (Kim & Kim 2011; Kim *et al*. 2012) and for the host dynamics of SVCV infection (Liu *et al*. 2013).

## Conclusion

According to our results, knock-down by synthetic siRNA of phosphoprotein or nucleoprotein reduces viral particle release from an *epithelioma papulosum cyprini* cell line infected with SVCV. The use of siRNAs in tandem, which target phosphoprotein and nucleoprotein, works best in reducing *in vitro* viral replication. In addition, two sets of primers were developed to measure SVCV-N or SVCV-P mRNA transcription.
